# Functional dissection of hematopoietic stem cell populations with a stemness-monitoring system based on NS-GFP transgene expression

**DOI:** 10.1038/s41598-017-11909-3

**Published:** 2017-09-12

**Authors:** Mohamed A. E. Ali, Kyoko Fuse, Yuko Tadokoro, Takayuki Hoshii, Masaya Ueno, Masahiko Kobayashi, Naho Nomura, Ha Thi Vu, Hui Peng, Ahmed M. Hegazy, Masayoshi Masuko, Hirohito Sone, Fumio Arai, Atsushi Tajima, Atsushi Hirao

**Affiliations:** 1 Division of Molecular Genetics, Cancer Research Institute, Kanazawa University, Kanazawa, Japan; 2 Department of Hematology, Endocrinology and Metabolism, Faculty of Medicine, Niigata University, Niigata, Japan; 3 Department of Stem Cell Biology and Medicine, Faculty of Medical Sciences, Kyushu University, Kyushu, Japan; 4 Department of Bioinformatics and Genomics, Graduate School of Advanced Preventive Medical Sciences, Kanazawa University, Kanazawa, Japan

## Abstract

Hematopoietic stem cells (HSCs) in a steady state can be efficiently purified by selecting for a combination of several cell surface markers; however, such markers do not consistently reflect HSC activity. In this study, we successfully enriched HSCs with a unique stemness-monitoring system using a transgenic mouse in which green florescence protein (GFP) is driven by the promoter/enhancer region of the nucleostemin (NS) gene. We found that the phenotypically defined long-term (LT)-HSC population exhibited the highest level of NS-GFP intensity, whereas NS-GFP intensity was strongly downregulated during differentiation *in vitro* and *in vivo*. Within the LT-HSC population, NS-GFP^high^ cells exhibited significantly higher repopulating capacity than NS-GFP^low^ cells. Gene expression analysis revealed that nine genes, including Vwf and Cdkn1c (p57), are highly expressed in NS-GFP^high^ cells and may represent a signature of HSCs, i.e., a stemness signature. When LT-HSCs suffered from remarkable stress, such as transplantation or irradiation, NS-GFP intensity was downregulated. Finally, we found that high levels of NS-GFP identified HSC-like cells even among CD34^+^ cells, which have been considered progenitor cells without long-term reconstitution ability. Thus, high NS-GFP expression represents stem cell characteristics in hematopoietic cells, making this system useful for identifying previously uncharacterized HSCs.

## Introduction

Hematopoietic stem cells (HSCs) perpetuate undifferentiated status through self-renewal, but also give rise to progenitors, which no longer possess the capacity for self-renewal but can differentiate into committed lineages^1^. Accumulating evidence supports the presence of “stemness” signals, that is, molecules and signaling pathways that play critical roles in maintaining the undifferentiated properties of HSCs. HSCs are maintained in a quiescent status in the bone marrow niches, and cooperative networks of intrinsic and extrinsic factors contribute to stem cell homeostasis *in vivo* ^2^. To date, numerous factors involved in microenvironments, the cell cycle, metabolism, and epigenetics have been identified as regulators of HSC homeostasis^3, 4^; however, it remains unclear how the stemness is regulated to maintain homeostasis.

The identification of an HSC population is based on differential expression of cell surface markers. Positive selection for c-Kit (a receptor for stem cell factor; also known as CD117) and Sca-1 in combination with negative selection of mature hematopoietic markers (Lineage markers) efficiently enrich HSC/progenitor cells (Lineage^−^Sca-1^+^c-Kit^+^; LSK)^5^. Additional markers, including CD34, CD150, Flt3, and CD48, have been used to isolate HSCs that have long-term (LT) reconstitution activity (LT-HSCs, e.g. CD150^+^CD48^−^CD34^−^ LSK cells)^6, 7^. In the classical model of hematopoietic hierarchy, LT-HSCs are distinguished from multipotent progenitors (MPP), common myeloid progenitors (CMP), common lymphoid progenitors (CLP), granulocyte and macrophage progenitors (GMP), and megakaryocyte and erythroid progenitors (MEP)^8, 9^. However, several studies have revealed novel concepts regarding HSC homeostasis that are distinct from the classical hierarchy^10^. Recent studies using transcriptomic, proteomic, and epigenetic profiling of HSCs have revealed that HSC populations are heterogeneous^11^, and studies with additional indicators have demonstrated that even the LT-HSC population is heterogeneous^12^. Therefore, additional tools are required to further separate HSCs into their component populations.

Previously, we attempted to identify a novel stem cell population by using the promoter/enhancer region of nucleostemin (NS, also known as Gnl3). NS encodes a GTP-binding protein that was first discovered in neural stem cells^13^. The expression of NS is reduced considerably during neural stem cell differentiation both *in vitro* and *in vivo*. Furthermore, overexpression of NS causes reprogramming of somatic cells into induced pluripotent stem cells^14^. In the hematopoietic system, NS protects HSCs from genotoxic insults and subsequent apoptosis, thereby maintaining HSC function, although NS has been reported to be highly expressed in hematopoietic progenitor cells as well as HSCs^15^. To monitor NS expression *in vivo*, we generated a transgenic mouse in which green florescence protein (GFP) is driven by the promoter/enhancer region of the NS gene (NS-GFP transgenic mouse). Using this system, we were able to isolate the stem cell populations in neonatal testicular cells, liver cells, and foetal brain tissues^16–18^. In addition, we identified tumour-initiating cells in mouse models of brain tumours and germ cell tumours^18, 19^. NS is also reported to be associated with poor prognosis in patients with acute myeloid leukaemia (AML) because it is closely correlated with immature blast cell percentage and stem cell surface markers such as CD34^20^. Moreover, we previously reported that AML stem cells reside in a rare population of leukaemia cells that highly express NS-GFP in our system, and these cells had the ability to initiate leukaemia upon transplantation into recipient hosts^20^. In that study, we identified a leukaemia stem cell gene signature that could be used to categorize AML patients into distinct prognostic groups.

In this study, we evaluated the NS-GFP expression pattern in normal hematopoiesis in the NS-GFP transgenic mice. We found that NS-GFP intensity was well correlated with the repopulating capacity of HSCs, and this system was useful for identifying previously unrecognized HSCs. Further dissection of HSCs by using NS-GFP may lead to a deeper understanding of the nature of the stem cell system *in vivo*.

## Results

### Tight association of NS-GFP intensity with differentiation process in multilineage hematopoiesis

**Figure 1:**
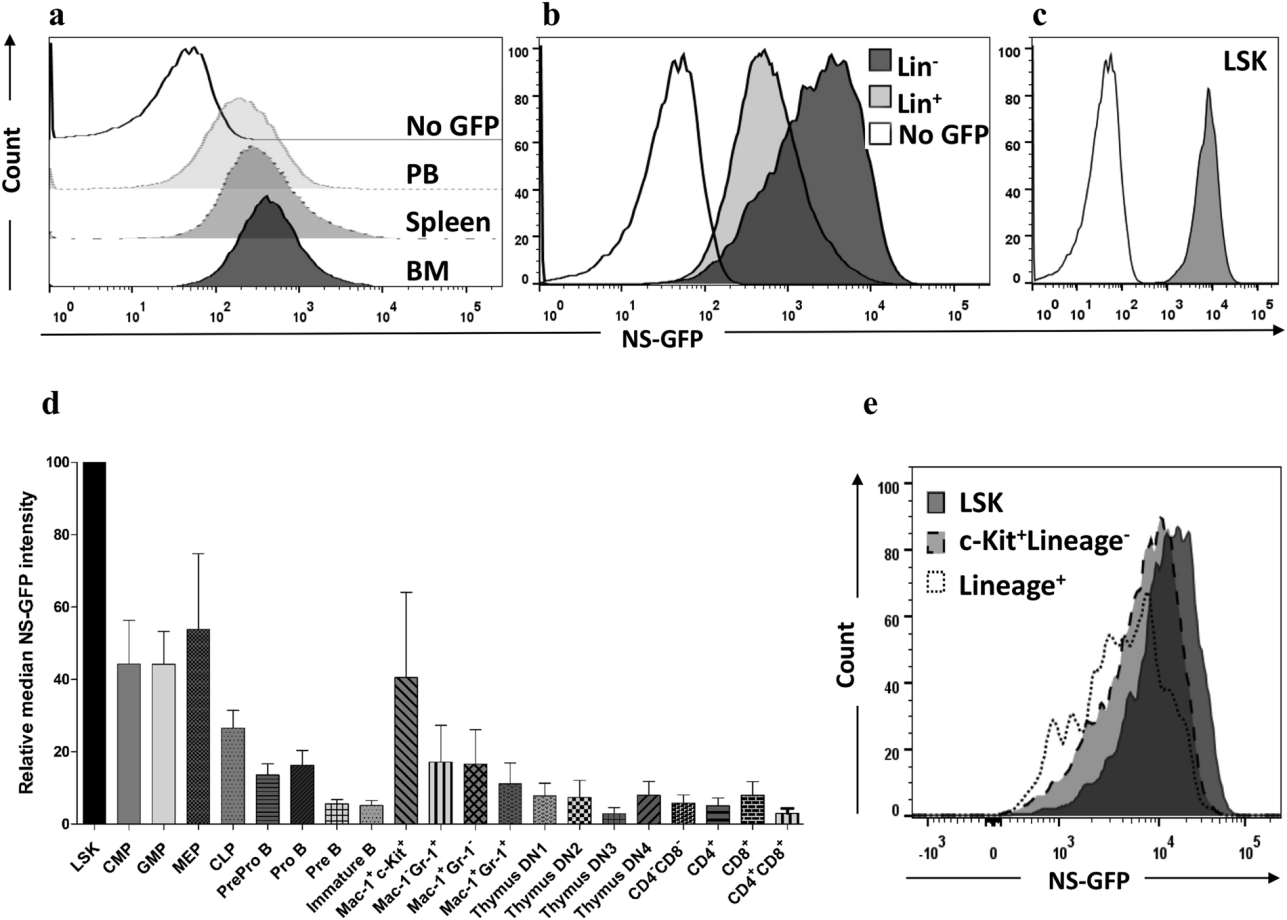
NS-GFP intensity reflects differentiation status in the hematopoietic system. FACS analysis of NS-GFP intensity in (**a**) bone marrow (BM), spleen, and peripheral blood (PB) mononuclear cells (MNCs), (**b**) Lineage (Lin)^−^ and Lin^+^ cells, (**c**) Lin^−^Sca-1^+^c-Kit^+^ (LSK) cells, and (**d**) differentiated cell lineages (CMP, common myeloid progenitors; GMP, granulocyte and macrophage progenitors; MEP, megakaryocyte and erythroid progenitors; CLP, common lymphoid progenitors). Data shown are the average ratios ± SD of median NS-GFP intensity of individual subpopulation, relative to LSK cells (n = 3). (**e**) *In vitro* differentiation of LSK cells showing downregulation of NS-GFP intensity upon expression of lineage markers.

First, we analyzed the NS-GFP intensity of cells in bone marrow (BM), spleen, and peripheral blood (PB) (Fig. 1a). The intensity of the NS-GFP fluorescence appeared to ascend gradually from PB to spleen and finally to BM. In BM, most mature cells, including myeloid cells (Mac-1^+^ and/or Gr-1^+^), B cells (B220^+^), T cells (CD4^+^ and/or CD8^+^), erythroid cells (Ter119^+^), and megakaryocytes (CD41^+^) showed lower intensity than Lineage^−^ cells, which represent an undifferentiated cell population (Fig. 1b–d). Moreover, LSK cells, which include both HSCs and MPPs (together known as HSPCs), showed the highest expression among BM cells (Fig. 1c). In the myeloid lineage, LSK cells differentiate into CMPs, then GMPs, and then Mac-1^+^ cells. NS-GFP intensity was gradually downregulated at each stage of differentiation from MPP to Mac-1^+^ cells. Among myeloid-committed cells, immature myeloid cells (c-Kit^+^ Mac-1^+^) expressed relatively higher levels of NS-GFP than more-differentiated myeloid cells, including granulocytes (Mac-1^+^Gr-1^+^). In the B cell lineage, we found that the Pre-Pro B cell and Pro B cell stages had relatively higher fluorescence intensities than Pre B cells and immature B cells. Most T cells in the thymus and BM expressed low levels of NS-GFP (Fig. 1d). Thus, the NS-GFP intensity is tightly associated with the differentiation process in multilineage hematopoiesis, with undifferentiated cells expressing relatively higher levels of NS-GFP than more-differentiated cells.

To investigate the relationship between NS-GFP activity and differentiation *in vitro*, LSK cells were cultured *in vitro* with serum, interleukin (IL)-3, and IL-6 to initiate cellular differentiation. Analysis of cultured cells after 2 days revealed that LSK cells lost NS-GFP intensity during differentiation (Fig. 1e), confirming the relationship between NS-GFP intensity and hematopoietic differentiation status.

### NS-GFP intensity is highest in LT-HSCs

**Figure 2:**
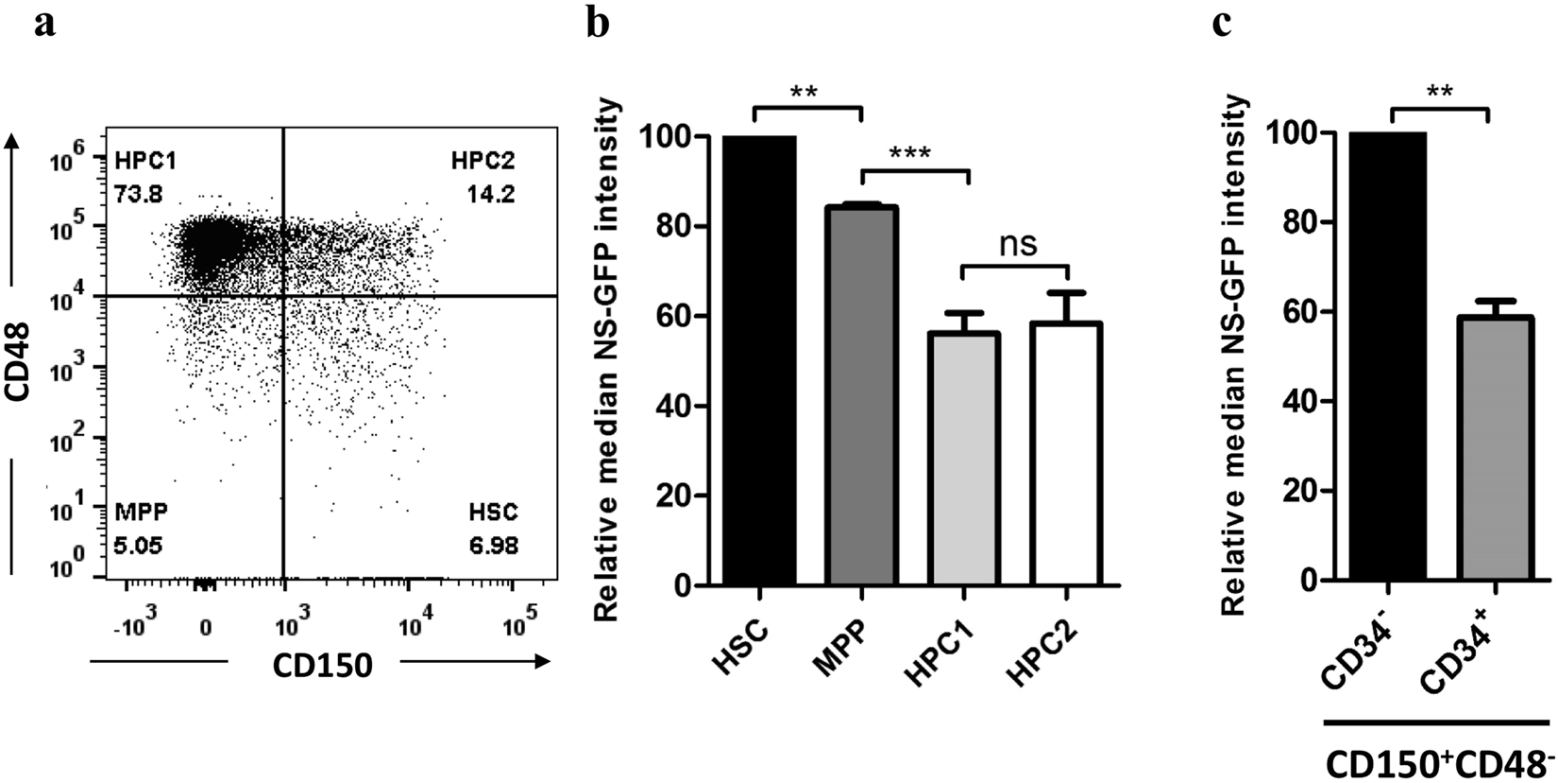
SLAM markers identify LT-HSCs that show the highest NS-GFP intensity. (**a**) Identification of HSCs using the CD150 and CD48 staining profile of Lin^−^Sca-1^+^c-Kit^+^ bone marrow cells. (**b**) The highest NS-GFP intensity was detected in the HSC population, with gradual decline in multipotent progenitors (MPP) and restricted progenitors (HPC1 and HPC2). (**c**) Among CD150^+^CD48^−^ LSK cells, NS-GFP intensity is higher in CD34^−^ than in CD34^+^ cells. Data shown are the average ratios ± SD of median NS-GFP intensity of individual subpopulation, relative to HSCs in (**b**) and CD34^−^ cells in (**c**), respectively (n = 3). **P < 0.01; ***P < 0.001; ns, not significant.

Next, we evaluated NS-GFP intensity among LSK cells as HSPCs. LSK cells can be subfractionated, based on their expression of SLAM family markers (i.e., CD150 and CD48), into LT-HSCs (HSC: CD150^+^CD48^−^LSK), MPP (CD150^−^CD48^−^LSK), and restricted progenitors (HPC1: CD150^−^CD48^+^LSK and HPC2: CD150^+^CD48^+^LSK). The LT-HSC population showed the highest NS-GFP intensity of these progenitor cell populations (Fig. 2a,b). Because another important indicator of LT-HSCs is CD34, we compared NS-GFP intensity between CD150^+^CD48^−^ CD34^−^LSK cells and CD150^+^CD48^−^ CD34^+^LSK cells. Although both populations showed high levels of NS-GFP, the intensity of NS-GFP in CD150^+^CD48^−^CD34^−^LSK cells was higher than that in CD150^+^CD48^−^CD34^+^LSK cells (Fig. 2c). Thus, the level of NS-GFP expression corresponds with the expression of previously described HSC markers.

**Figure 3:**
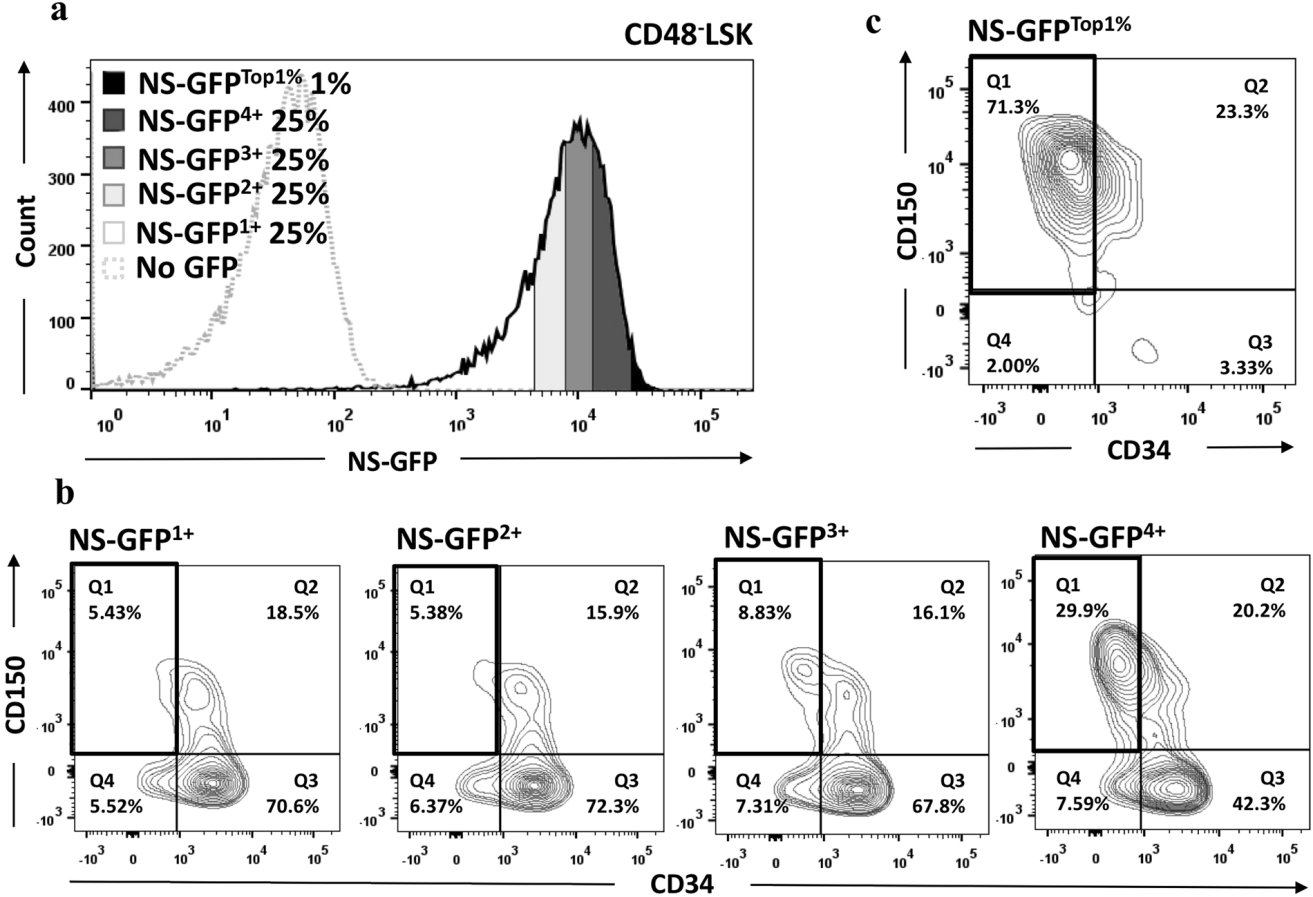
Enrichment of LT-HSCs by gating CD48^−^LSK cells on the highest NS-GFP intensity. (**a**) Fractionation of bone marrow CD48^–^LSK cells from an NS-GFP transgenic mouse into four equal fractions of NS-GFP intensity, plus the 1% of cells with the highest NS-GFP intensity. (**b**,**c**) CD150 and CD34 staining profiles of the LSK fractions with different NS-GFP intensity.

As an alternative analysis of LSK cells, we divided LSK (CD48^−^) cells into four fractions based on NS-GFP intensity (NS-GFP^1+^, NS-GFP^2+^, NS-GFP^3+^, NS-GFP^4+^) and evaluated the frequency of expression of two important markers, CD150 and CD34 (Fig. 3a,b). NS-GFP^4+^ (top 25% in GFP intensity) included more LT-HSCs (CD150^+^CD34^−^) than other populations, suggesting that HSCs are highly enriched in cell populations with higher levels of NS-GFP. This finding was apparent when we observed NS-GFP^Top1%^ cells (Fig. 3c). This provides further evidence that the highest NS-GFP intensity represents more phenotypically undifferentiated characteristics.

### HSC gene signature associated with NS-GFP intensity in HSPCs

We next examined whether NS-GFP intensity reflects endogenous NS mRNA. First, we analyzed NS mRNA among BM mononuclear (MNCs) cells fractionated according to NS-GFP intensity (Supplementary Fig. 1a). As expected, we found that both GFP and NS mRNA were correlated with NS-GFP intensity (Supplementary Fig. 1b,c). However, when we evaluated fractionated LSK cells (Supplementary Fig. 1d), GFP mRNA levels were well correlated with GFP intensity (Supplementary Fig. 1e), but NS mRNA levels were not (Supplementary Fig. 1f), indicating a discrepancy between the expression of GFP and endogenous NS in LSK cells. These data suggest that NS mRNA expression may be controlled by a complex regulatory machinery, including not only the promoter/enhancer region used for the NS-GFP transgenic mice, but also other regions that negatively control NS mRNA expression. However, although NS-GFP expression may be artificially induced by the transgene, it may be an indicator of functional HSCs.

**Figure 4:**
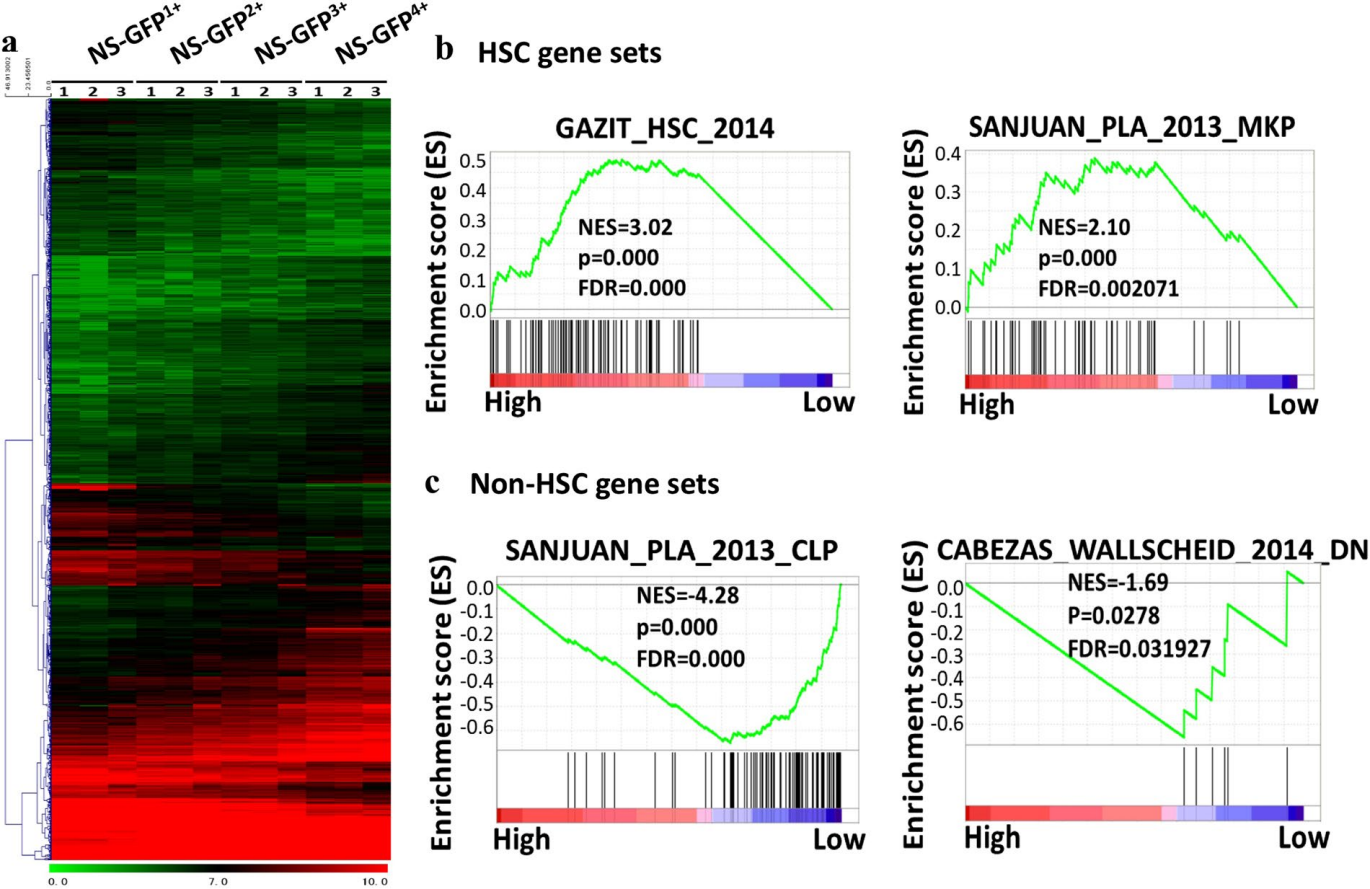
Gene set enrichment analysis of subpopulations of LSK cells. (**a**) Expression profiling of 1336 probes whose expression gradually increased or decreased along with the progressive changes among the four subpopulations (NS-GFP^1+^ to NS-GFP^4+^) (n = 3). (**b**,**c**) Representative data showing enrichment of “HSC gene sets” (**b**) and less enrichment of “non-HSC gene sets” (**c**) in NS-GFP^4+^ LSK cells.

To characterize the gene signature of hematopoietic cell subpopulations, we performed a microarray analysis using LSK cells subfractionated based on NS-GFP intensity (NS-GFP^1+^ to NS-GFP^4+^). We selected 1336 probes whose expression gradually increased or decreased along with the progressive changes in NS-GFP intensity among the four subpopulations, as described in the Materials and Methods (Fig. 4a, Supplementary Table 1 A,B). Among the probes that showed the largest difference between NS-GFP^4+^ and NS-GFP^1+^ LSK cells, we found numerous genes which had been reported as genes highly expressed in HSCs compared to progenitor cells/differentiated cells, including Vwf, Cdkn1c (p57), Kazald1, Egr1, Fgfr3, Mapk12, Hoxb5, Slc16a9, Aldh1a1, HoxB2, Tgm2, Obsl1, HoxB6, Wfdc2, Jam3, Sult1a1, Pdgfd, Meg3, Klhl13, and Gbp8^21–23^. Using these 1336 probes, we performed gene set enrichment analysis (GSEA) with experimentally determined “HSC gene sets” or “non-HSC gene sets”^21–23^. The genes upregulated in NS-GFP^4+^ were enriched in the “HSC gene sets” and less enriched in the “non-HSC gene sets” (Fig. 4b,c). In an additional GSEA with the “chemical and genetic perturbations (CGP)” category (see the Methods), we found that 15 gene sets were upregulated in NS-GFP^4+^, including “IVANOVA_HEMATOPOIESIS_STEM_CELL_LONG_TERM” and “RAMALHO_STEMNESS_UP”, and 36 gene sets were downregulated, including “HADDAD_B_LYMPHOCYTE_PROGENITOR”, “JAATINEN_HEMATOPOIETIC_STEM_CELL_DN”, and “IVANOVA_HEMATOPOIESIS_LATE_PROGENITOR” (FDR [false discovery rate] < 0.25, nominal-*p* < 0.01) (Supplementary Table 2). These results suggest that NS-GFP^4+^ cells have many more HSC characteristics than NS-GFP^1+^ cells, again confirming that NS-GFP expression is well correlated with the gene signature of HSC differentiation.

### Purification of cells with higher repopulating capacity within phenotypically defined LT-HSCs

**Figure 5:**
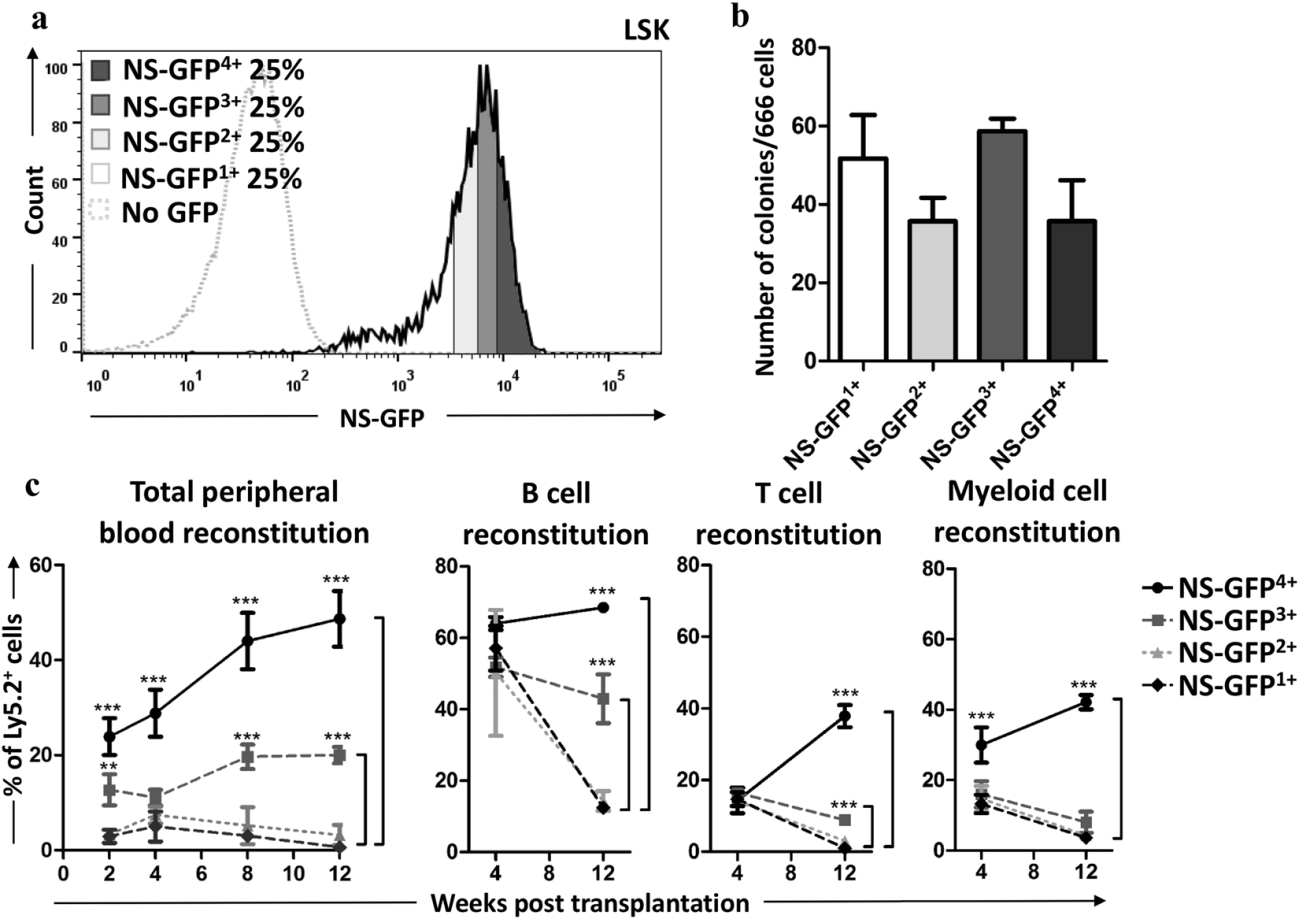
Repopulation capacity of the HSPC populations with different NS-GFP intensity. (**a**) FACS pattern of bone marrow LSK separation into four fractions according to NF-GFP intensity. (**b**) An *in vitro* colony formation assay shows no clear difference between the four LSK fractions. (**c**) After transplantation of the four fractions into lethally irradiated hosts (1,000 cells were transplanted per mouse), NS-GFP ^4+^ had the highest reconstitution capacity with multilineage differentiation potential. Data shown are the mean frequencies of Ly5.2^+^ cells in the peripheral blood and the mean frequencies of Ly5.2^+^ cells among B cells, T cells or myeloid cells ± SD (n = 3). **p < 0.01; ***p < 0.001.

To investigate whether NS-GFP activity is a marker of HSCs, we divided LSK cells into four subpopulations according to NS-GFP intensity (Fig. 5a), then subjected these subpopulations to colony-forming assays and transplantation into lethally irradiated mice. Although all subfractionated cells had comparable colony-forming ability *in vitro* (Fig. 5b), cells expressing less GFP (NS-GFP^1+^ and NS-GFP^2+^) did not have long-term reconstitution capacity (Fig. 5c), indicating that most of these cells are progenitors. Cells expressing higher levels of GFP (NS-GFP^3+^ and NS-GFP^4+^) showed greater repopulating capacity, but the frequency of NS-GFP^4+^-derived hematopoietic cells was much higher than that of NS-GFP^3+^-derived cells. Differentiation marker analysis showed that only NS-GFP^4+^ produced B cells, T cells, and myeloid lineage cells (Fig. 5c), although the colony-forming abilities of NS-GFP^4+^ and NS-GFP^3+^ cells were comparable. Thus, the NS-GFP^4+^ subpopulation highly enriched cells with greater repopulating capacity, suggesting that NS-GFP expression can be used to purify LT-HSCs.

**Figure 6:**
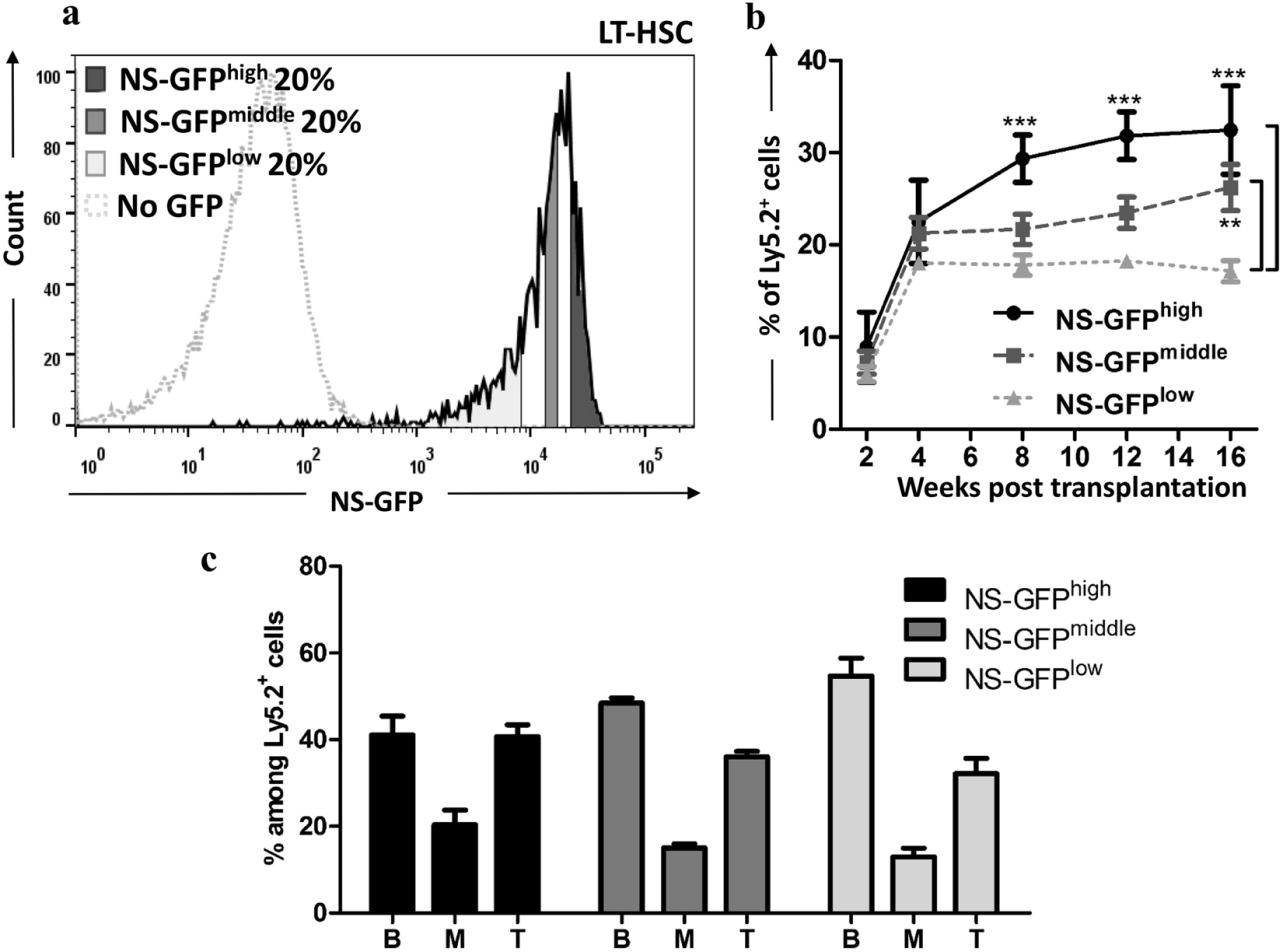
Repopulation capacity of phenotypically defined LT-HSCs. (**a**) FACS was used to separate bone marrow LT-HSCs into five fractions according to NS-GFP intensity, of which three fractions—the top 20% (GFP^high^), middle 20% (GFP^middle^), and bottom 20% (GFP^low^)—were used in subsequent experiments. (**b**) The LT-HSC fractions show significant differences in reconstitution capacity after transplantation into lethally irradiated hosts. Ten cells were transplanted per mouse. Data shown are the mean frequencies of Ly5.2^+^ cells in the peripheral blood ± SD (n = 5). (**c**) Regeneration in multilineages derived from fractionated LT-HSCs in the peripheral blood of recipient mice after 16 weeks. Data shown are the mean frequencies of B cells, T cells or myeloid cells among Ly5.2^+^ cells ± SD (n = 5). B; B cells. M; myeloid cells. T; T cells. **p < 0.01; ***p < 0.001.

CD150^+^CD48^−^CD34^−/low^ LSK cells are the most established phenotypically defined LT-HSCs as evaluated by transplantation^6^. We investigated whether we could use NS-GFP expression to enrich an even more purified population of HSCs. We fractionated CD150^+^CD48^−^CD34^−/low^ LSK cells into three populations based on NS-GFP intensity (NS-GFP^high^, NS-GFP^middle^, and NS-GFP^low^) (Fig. 6a). We transplanted 10 cells isolated from each fraction into lethally irradiated mice. Although the three fractions were indeed capable of long-term reconstitution of the BM in recipient mice, the frequencies of donor-derived cells among the three fractions were significantly different. NS-GFP^high^ cells showed the highest reconstitution capacity, whereas NS-GFP^low^ showed the lowest (Fig. 6b). All fractions showed comparable multilineage differentiation potential (Fig. 6c). Thus, NS-GFP expression allowed us to further enrich cells with higher repopulating capacity.

### Single-cell analysis of LT-HSCs sorted by NS-GFP intensity

**Figure 7:**
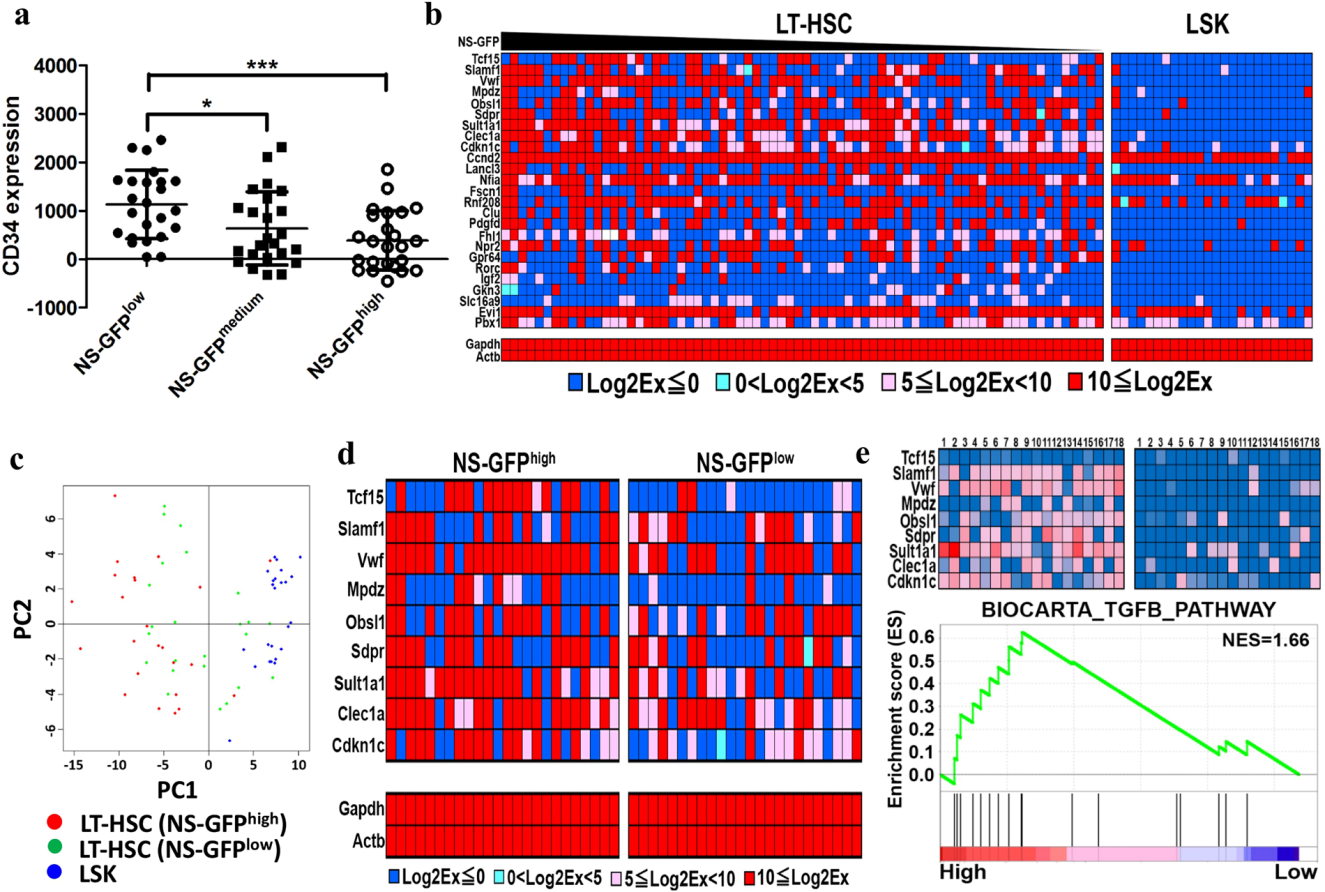
Single-cell analysis of HSCs characterized with the NS-GFP monitoring system. (**a**) Single-cell analysis of LT-HSCs sorted according to NS-GFP intensity, showing significant differences in CD34 expression, data shown are the mean intensity of CD34 expression ± SD (n = 24). *p < 0.05; ***p < 0.001. (**b**) Single-cell qPCR of 25 genes with differential expression between LT-HSC and LSK cells. (**c**) Principal component analysis of fractionated LT-HSC (NS-GFP^high^ and NS-GFP^low^) and HSPC cells (LSK). (**d**) Stemness gene signature differentially expressed between NS-GFP^high^ and NS-GFP^low^ LT-HSC. (**e**) A single-cell RNA sequencing data set was used to rank LT-HSCs according to the expression of stemness gene signature (top left panel, “Top 20%”; top right panel, “Bottom 20%”), followed by GSEA (bottom panel) that shows the TGF-β pathway to be highly enriched in LT-HSCs expressing the stemness gene signature (nominal-*p* = 0.006).

To examine the relationship among the expression of NS-GFP, surface markers (protein), and genes (mRNA) within individual LT-HSCs, we performed index sorting of LT-HSCs followed by single-cell qPCR with primers for HSC-specific genes. We found significant differences in CD34 expression among the three groups (NS-GFP^low^, NS-GFP^medium^, and NS-GFP^high^) even when we analyzed LT-HSCs sorted as CD34^−/low^ cells (Fig. 7a). Because downregulation of CD34 is a hallmark of HSCs, it was suggested that HSCs are highly enriched in NS-GFP^high^ cells, which had low CD34 content. This is also consistent with the repopulation assay (Fig. 6b). For single-cell qPCR, among selected primer sets of over 100 genes that have been reported to be more highly expressed in LT-HSCs than in HSPCs, we found that 25 genes showed a significant difference in expression level or frequency between LT-HSCs and HSPCs (LSK) (Fig. 7b). Then, using primers for these 25 genes, we performed single-cell PCR of NS-GFP^high^ and NS-GFP^low^ LT-HSCs. A principal component analysis indicated that NS-GFP^high^ LT-HSCs are distinct from HSPCs (LSK) and that NS-GFP^low^ LT-HSCs fall between them (Fig. 7c), suggesting that NS-GFP^high^ LT-HSCs are more primitive than NS-GFP^low^ LT-HSCs. Among the 25 genes, nine genes (*Tcf15*, *Slamf1*, *Vwf*, *Mpdz*, *Obsl1*, *Sdpr*, *Sult1a1*, *Clec1a*, *and Cdkn1c* [*p57*]) showed higher frequency in NS-GFP^high^ than in NS-GFP^low^ LT-HSCs (Fig. 7d), suggesting that LT-HSCs are a heterogeneous population and that combinations of these genes determine the functional properties of HSCs.

Recently, several groups have performed single-cell RNA sequencing of LT-HSCs, and their data are publically available. Using the data of 86 LT-HSCs that have undergone quality control^24^, we attempted to rank the LT-HSCs based on expression levels of the nine stemness genes among the individual cells (see the Materials and Methods). From the 86 LT-HSCs, we selected 18 cells (Fig. 7e) that fell into the top 20% and 18 that fell into the bottom 20% of expression of the nine stemness genes and compared gene set expression between the two groups (Fig. 7e). GSEA indicated that a curated TGF-β pathway (BIOCARTA_TGFB_PATHWAY) displayed the highest absolute normalized enrichment score (NES) (NES = 1.66), and this pathway was significantly enriched at nominal-*p* = 0.006 in the “Top 20%“cells compared with the “Bottom 20%” cells (Fig. 7e). Unfortunately, this enrichment did not achieve a more stringent level of significance (FDR < 0.25), presumably due to the small number of cells used for the analysis, but this finding suggests that TGF-β signals may play a critical role in the stemness of HSCs.

### Identification of uncharacterized HSCs by NS-GFP

**Figure 8:**
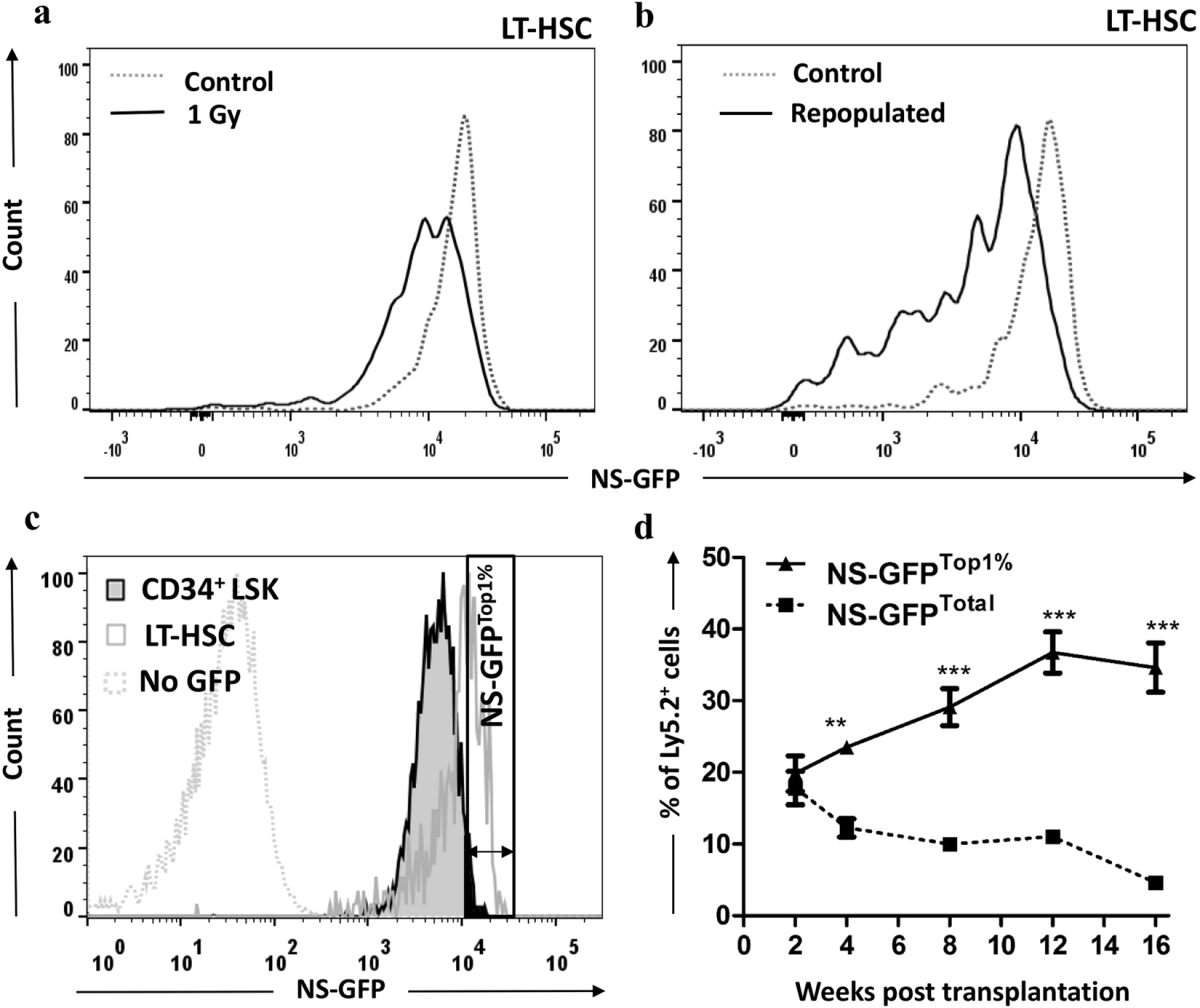
Identification of a novel cell population with high repopulating capacity among cells that are phenotypically progenitor cells. (**a**) Stress-induced downregulation of NS-GFP intensity after 1 Gy irradiation to LT-HSCs. (**b**) LT-HSCs that repopulated lethally irradiated hosts for 16 weeks have lower NS-GFP intensity than controls. (**c**) FACS isolation of bone marrow CD34^+^ LSK with NS-GFP^Top1%^ intensity in relation to NS-GFP^Total^. (**d**) CD34^+^ LSK cells with the Top 1% of NS-GFP intensity show greater long-term reconstitution capacity than the total population of cells expressing NS-GFP. Fifty cells were transplanted per mouse. Data shown are the mean frequencies of Ly5.2^+^ cells in the peripheral blood ± SD (n = 5). **p < 0.01; ***p < 0.001.

Although the phenotypically defined LT-HSCs efficiently enriched functional HSCs, previous studies demonstrated that these surface molecules may not be efficient HSC markers under conditions of stress. For example, the ability of phenotypic LT-HSCs to repopulate BM is dramatically reduced by irradiation^25^. Similarly, when a donor-derived LT-HSC population appears after transplantation (regeneration of HSCs), these LT-HSCs show much less capacity for regeneration than the pretransplantation population, judging by secondary transplantation^26^. We found that a low dose of irradiation (1 Gy) reduced the NS-GFP intensity in LT-HSCs (Fig. 8a). We confirmed that the NS-GFP intensity reflects the repopulating capacity of irradiated (Supplementary Fig. 2) as well as normal HSCs. In addition, when BM MNCs from an NS-GFP transgenic mouse were allowed to repopulate lethally irradiated host mice for 16 weeks, we observed a clear reduction in NS-GFP intensity in donor-derived LT-HSC (Fig. 8b). These data suggest that NS-GFP intensity, rather than cell surface markers, accurately reflects the ability of damaged HSCs to repopulate host BM.

Finally, we attempted to identify HSCs within a cell population that has been considered a non-HSC population. As expected, we did not see repopulation when we transplanted 50 CD34^+^ LSK cells, a population of MPPs that do not have long-term reconstitution ability. We then sorted 50 NS-GFP^Top1%^ cells from CD34^+^ LSK cells (Fig. 8c) and transplanted them into irradiated recipient mice. Interestingly, NS-GFP^Top1%^ CD34^+^ LSK cells showed strong reconstitution ability (Fig. 8d). Consistent with this, transplanted CD34^+^LSK fractions that expressed levels of NS-GFP comparable with NS-GFP^high^ LT-HSCs showed high repopulating capacity with multilineage differentiation potential, like HSCs (Supplementary Fig. 3). Although we do not exclude a possibility that there may be functional differences between these populations, because we did not investigate the repopulating capacity over a longer time or by serial transplantation, these data clearly demonstrate that the NS-GFP system allow us to identify such HSC-like cells among CD34^+^ cells.

## Discussion

Immunophenotypically defined populations of HSCs have been suggested to be functionally heterogeneous, differing with respect to various properties including cell cycle status, engraftment capacity, lineage bias, and self-renewal potential. For example, a recent study of single-cell transplantation unexpectedly discovered the presence of myeloid-restricted progenitors with long-term repopulating activity^27^, which contradicts the classical HSC hierarchical model. Another study using von Willebrand factor (vWF)-GFP reporter mice, in which the expression level of vWF was monitored, showed that HSCs with active platelet-specific genes have a long-term myeloid lineage bias, but can self-renew and produce lymphoid-biased HSCs after self-renewal. Thus, these platelet-biased HSCs are at the top of the HSC hierarchy^21^. Furthermore, stem-like megakaryocyte-committed progenitors, which serve as a lineage-restricted emergency pool for responding to inflammatory insults, have been identified^28, 29^. These data suggest that the HSC hierarchal organization is more complicated than previously assumed and presumably can be dramatically affected by changes in the microenvironment. In addition, although most HSCs are efficiently enriched by selection as phenotypic LT-HSCs (i.e., CD150^+^CD48^–^CD34^−^LSK cells) at a steady state, these surface molecules may not necessarily be efficient HSC markers under stresses such as chronic inflammation, genotoxicity, and aging. For example, when BM cells were regenerated in recipient mice after transplantation, donor-derived HSCs dramatically lost their repopulating capacity despite forming a phenotypical HSC population, indicating that there is a discrepancy between phenotypes (i.e., surface markers) and their functions. In a similar way, the number of phenotypical HSCs is remarkably increased in aged mice, yet the number of functional HSCs appears to be reduced^26^. These findings indicate that HSC markers are not sufficient to allow us to enrich functional HSCs that have been exposed to stress. Therefore, we believe that our NS-GFP system contributes to identifying uncharacterized stem cell populations.

The NS-GFP system allowed us to enrich HSCs with higher repopulating capacity, as shown in Fig. 7. Interestingly, gene expression patterns of the nine stemness genes that we identified were not homogenous (Fig. 7d). Although all nine of the genes are highly expressed in LT-HSCs, these genes are not expressed simultaneously in the same cells, and only a small population among the NS-GFP^high^ LT-HSCs expressed all of these genes. This finding indicates that combinations of these genes, rather than a single gene, may be important for the determination of HSC properties, leading to functional heterogeneity. Our analysis of combinatory gene expression analysis in single cells raised the possibility that the TGF-β pathway plays an important role in regulating stemness. As an additional application, this system may be useful for identifying previously uncharacterized HSCs, because we found that cells with higher NS-GFP expression in the MPP compartment showed high repopulating capacity. This may contribute to more detailed understanding of the HSC hierarchy.

When we analyzed the endogenous NS mRNA in fractionated LSK cells, we did not find a significant difference among the different groups, although a clear functional difference was evident in the long-term reconstitution experiment, indicating a discrepancy between endogenous NS mRNA and NS-GFP. This result was consistent with a previous report showing that endogenous NS mRNA was highly expressed in immature hematopoietic cells such as MEPs, GMPs, CMPs, MPPs, and HSCs, but the expression is similarly high among these cell populations^15^. The exact reason why NS-GFP labels HSCs is unclear; however, it is assumed that the promoter/enhancer that drives GFP expression in this transgenic mouse may include an important region for stem-cell-specific expression. The region 7 kb upstream of the NS gene transcription start site that was used for this transgenic mouse contains CpG islands and possible methylation sites. A recent genome-wide analysis of DNA methylation reported that there are changes of methylation status in the genome that are specific for HSCs, for example in the HoxB family loci. Because HoxB family genes are highly expressed in HSCs, such epigenetic modifications are assumed to be involved in HSC-specific upregulation of HSC-specific genes. In addition, it is possible that chromatin modification, e.g., histone methylation, may control the expression of GFP in the transgenic mouse. Therefore, such epigenetic changes in the promoter/enhancer region of NS may control GFP expression, whereas endogenous NS mRNA expression may be modified by multiple factors, such as RNA stabilization, in addition to epigenetic regulation. Alternatively, negative inhibitory loops in complex regulatory pathways may result in the discrepancies we saw between NS-GFP promoter activity and endogenous NS mRNA levels. Moreover, in our previous study of NS-GFP, we did not find any correlation between endogenous NS mRNA levels and patient prognosis, but we successfully identified a gene set associated with a highly active NS promoter that clearly reflected a significant difference in patient prognosis^20^. Collectively, these results suggest that, although endogenous NS is not the key player in our finding, the NS promoter/enhancer by itself might represent a major regulatory influencer that orchestrates a wide range of critical factors associated with HSCs.

In conclusion, we successfully dissected the HSC population according to HSC repopulating capacity based on NS-GFP transgene expression and identified possible signals as critical regulators of HSC behavior. Because we found that the NS-GFP transgene is useful for identifying HSC-like cells with higher repopulating capacity in cell types other than HSCs, a comprehensive analysis using this NS-GFP system may reveal core mechanisms of universal stemness regulation.

## Materials and Methods

### Mice

All data presented in this study were obtained from experiments using heterozygous NS-GFP transgenic (tg) mice on a C57BL/6 background as described previously^16^. C57BL/6 (B6)-Ly5.1 or Ly5.2 WT mice were purchased from Sankyo Labo Service (Tsukuba, Japan). All animals were maintained at the Advanced Science Research Center Institute for Experimental Animals, Kanazawa University, according to guidelines approved by the Committee on Animal Experimentation of Kanazawa University, Japan. All animal experiments were approved by the Committee on Animal Experimentation of Kanazawa University and performed in accordance with the university’s guidelines for the care and use of laboratory animals.

### Bone marrow and peripheral blood collection

Total BM cells were obtained from femoral and tibial bones by aspiration. MNCs were isolated from total BM cells by density gradient centrifugation using Lymphoprep (Axis-Shield). Peripheral blood cells were collected from the postorbital vein and suspended in diluted heparin solution. MNCs in PB were isolated by dextran sedimentation and ammonium chloride lysis of erythrocytes as described previously^30^.

### Flow cytometry

Monoclonal antibodies against the following markers were used to recognize different hematopoietic cell populations: B220, CD4, CD8, Mac-1, Gr-1, Ter119, CD48, Sca-1, c-Kit, CD34, CD150, FcγIII/II receptor, IL-7Rα chain, CD19, CD43, IgM, CD44, CD25, CD41, CD45.1 (Ly5.1) and CD45.2 (Ly5.2) (all from BD Biosciences or eBiosciences) as previously described^30^. Marker analyses were performed using a FACSCanto II flow cytometer (BD Biosciences), and cell sorting was performed using a FACSAria cell sorter (BD Biosciences).

### Competitive repopulation assay *in vivo*

Tester cells from NS-GFP tg mice (Ly5.2) and competitor wild-type BM-MNCs (5 × 10^5^, Ly5.1) were intravenously transplanted into lethally irradiated (9.5 Gy) recipient mice (Ly5.1). Peripheral blood was collected at the indicated time points, and hematopoietic cells regenerated from tester cells were examined by flow cytometry.

### Irradiation-induced stress

NS-GFP tg mice were subjected to 1 Gy irradiation, and BM cells were collected for analysis 2 weeks later. BM cells were also collected from control, non-irradiated mice.

### *In vitro* liquid culture

Progenitor cells (c-Kit^+^ Lineage^−^, 1 × 10^4^) isolated from BM of NS-GFP tg mice were cultured for 2 days in RPMI 1640 containing 20% FBS, 10 ng/ml recombinant murine (rm)IL-3, and 10 ng/ml rmIL-6 at 37 °C in humidified air containing 5% CO_2_.

### Colony formation assay

LSK fractions isolated from NS-GFP tg mice (2 × 10^3^ cells each) were cultured for 7 days in semisolid medium containing 50 ng/ml recombinant murine stem cell factor (rmSCF), 10 ng/ml rmIL-3, 10 ng/ml rmIL-6, and 3 U/ml recombinant human erythropoietin (rhEPO) (Methocult GF M3434, Stem Cell Technologies) at 37 °C in humidified air containing 5% CO_2_.

### Quantitative RT-PCR analysis

RNA samples were purified from fractionated leukaemia cells (1 × 10^4^) using an RNeasy kit (QIAGEN) and reverse-transcribed using an Advantage RT-for-PCR kit (Clontech, Takara Bio Inc.). PCR for NS was performed using a Dice PCR Thermal Cycler (Takara Bio Inc.) as previously reported^16^.

### Statistics

Unless otherwise stated, statistical differences between two groups were determined using unpaired Student’s *t*-tests. Statistical differences between more than two groups were determined by one-way or two-way ANOVA with a Bonferroni post hoc test. *P-values* between indicated groups are shown in each figure.

### Microarray study design

Mouse LSK cells were sorted into four fractions: (a) NS-GFP^1+^ (lowest quartile of GFP intensity), (b) NS-GFP^2+^, (c) NS-GFP^3+^, and (d) NS-GFP^4+^ (highest quartile). Total RNA from 1 × 10^4^ cells in each fraction was isolated by using TRIzol (Invitrogen). Microarray experiments were carried out using three replicate RNA samples per fraction. Samples were processed by Hokkaido System Science Co., Ltd., and run on Agilent SurePrint G3 mouse gene expression arrays (single color). Expression data were processed with GeneSpring 12.6 GX by using quantile normalization with no baseline transformation. Probes with expression in the lowest 20% or which had detection calls of “0” or “1” were removed, and then 30104 of 42071 probes were extracted. The expression data were log_2_ transformed. To select probes whose expression changed progressively among the four subpopulations, we first extracted 1698 of 30104 probes showing |log_2_(NS-GFP^4+^ LSK/NS-GFP^1+^ LSK)| ≥ 1, then further selected 1336 probes that exhibited a gradual increase or decrease in expression that paralleled the progressive changes among the four subpopulations by matching either criteria 1, 2, and 3 or criteria 4, 5, and 6: (1) NS-GFP^4+^LSK ≥ NS-GFP^3+^LSK, (2) NS-GFP^3+^LSK ≥ NS-GFP^2+^LSK, (3) NS-GFP^2+^LSK ≥ NS-GFP^1+^LSK, (4) NS-GFP^4+^LSK ≤ NS-GFP^3+^LSK, (5) NS-GFP^3+^LSK ≤ NS-GFP^2+^LSK, (6) NS-GFP^2+^LSK ≤ NS-GFP^1+^LSK.

### Bioinformatics analysis

For microarray data, 1336 probes were analyzed with GSEA v2.2.0 (http://software.broadinstitute.org/gsea/index.jsp). These 1336 probes were annotated to 949 gene symbols during this analysis. In the first GSEA, we used a gene set published in three previous reports as related to the gene expression pattern of hematopoietic stem cells^21–23^. In the next GSEA, we used the “CGP category”, which represents the expression signatures of genes and chemical perturbations curated on Version 5.1 of the Molecular Signatures Database (MSigDB) (http://software.broadinstitute.org/gsea/msigdb/collections.jsp). The CGP category included 3396 gene sets. Gene set size filters (min = 10, max = 500) removed 2736 of the gene sets, and the remaining 660 were used in the analysis. Gene sets that showed FDR <0.25 and nominal-*p* < 0.01 (default setting) were considered to be statistically significant. For an extended analysis of nine stemness genes in LT-HSCs, a publicly available dataset of gene expression (expression count data for all genes/transcripts) in 96 individual LT-HSCs using single-cell RNA sequencing^24^ was obtained from the Gene Expression Omnibus database (accession number GSE61533). To remove putative low-quality cells from the dataset as a quality control measure, the median absolute deviation (MAD)-based method was applied to detect outlier cells that showed more than 3 MADs from the median with respect to (i) log-transformed library size (defined as the logarithm of the total counts across all genes), (ii) log-transformed number of expressed genes (defined as the logarithm of the number of genes with non-zero counts), (iii) the proportion of counts from mitochondrial genes, and (iv) the proportion of counts from External RNA Controls Consortium (ERCC) spike-in transcripts. After quality control on the cells, the remaining 86 LT-HSCs were used in the extended analysis. The sum of per-gene rankings based on single-cell expression levels of the respective nine stemness genes in the individual cells was employed to select 18 higher-ranking cells (corresponding to the “Top 20%”) and 18 lower-ranking cells (corresponding to the “Bottom 20%”) from the 86 LT-HSCs. To characterize global expression profiles from the “Top 20%” and “Bottom 20%” cells at the level of gene sets, GSEA was performed to search for candidate sets of genes correlated with the group distinction using the MSigDB curated pathways (BIOCARTA, KEGG, and REACTOME).

### Unsupervised hierarchical clustering and heatmap

Using MeV Ver.4.8.0 software, unsupervised hierarchical clustering was performed on 1386 probes to obtain the result shown in Fig. 4A. Similarity was measured by using a Euclidean distance metric and the complete linkage was used to define the linking distance between probes.

### Single-cell q-PCR

NS-GFP^high^, NS-GFP^medium^, or NS-GFP^low^ single cells in CD150^+^CD48^−^CD41^−^CD34^−^LSK or CD48^−^LSK single cells were index-sorted by FACS AriaII directly into 5-μL Reverse Transcription-Specific Target Amplification mix solution in a 96-well plate (BD Falcon 351177) to estimate the intensities of NS-GFP or CD34 expression in individual cells. cDNA was synthesized by using CellsDirect™ One-Step qRT-PCR Kit (Thermo Fisher Scientific). Pre-amplified samples (22 cycles) were diluted to 25 μL with Tris-EDTA buffer. TaqMan Universal PCR Master Mix (Applied Biosystems), TaqMan Gene Expression Assays (Applied Biosystems), 96.96 Dynamic Array, and BioMark System (Fluidigm) were used to perform PCR. *Actb* and *Gapdh* were used as internal controls. The BioMark Real-Time PCR Software (Fluidigm) was used for data analysis. TaqMan probes used are listed in Supplementary Table 3. Gene expression levels were estimated by subtracting the Ct values from the background level of 30, which approximates log_2_ gene expression levels. Ct values higher than 30 were transformed to 30 and were represented by zero in the data. To identify genes with significantly different levels of expression, the unpaired Student’s *t*-test, the Fisher’s Exact Test, and the chi-square test were used.

## Electronic supplementary material

Supplementary Figures

Supplementary Table 1A, 1B

Supplementary Table 2

Supplementary Table 3
