# Supplementary material for: Functional dissection of hematopoietic stem cell populations with a stemness-monitoring system based on NS-GFP transgene expression
Source: Sci Rep. 2017 Sep 12;7:11442. doi: 10.1038/s41598-017-11909-3 (PMC5596002; doi:10.1038/s41598-017-11909-3)
Supplement: Supplementary file 1 — Supplementary Figures [file 41598_2017_11909_MOESM1_ESM.pdf]

Functional dissection of hematopoietic stem cell populations with a stemness-monitoring system based on NS-GFP transgene expression

Mohamed A.E. Ali
Kyoko Fuse
Yuko Tadokoro
Takayuki Hoshii
Masaya Ueno
Masahiko Kobayashi
Naho Nomura
Ha Vu Thi
Hui Peng
Ahmed M. Hegazy
Masayoshi Masuko
Hirohito Sone
Fumio Arai
Atsushi Tajima
Atsushi Hirao

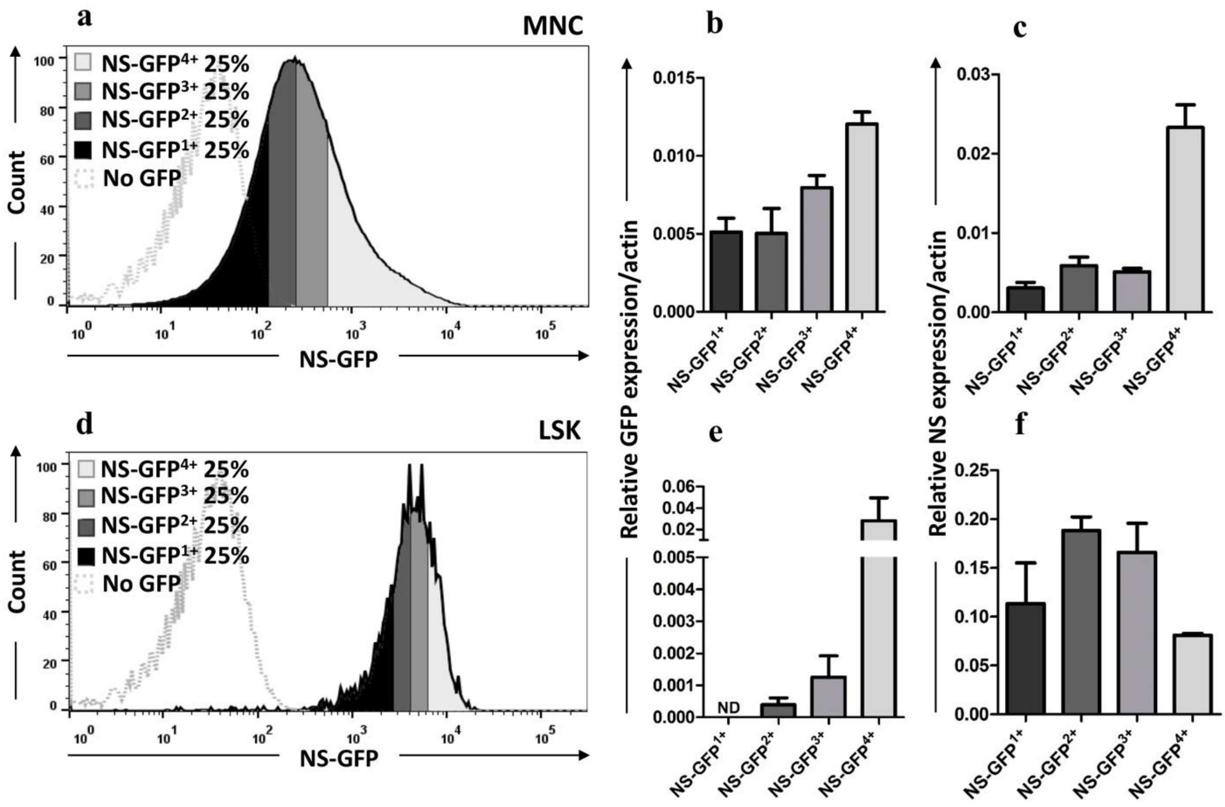

Supplementary Figure 1. Analysis of the correlation between NS-GFP and endogenous NS mRNA. (a) Bone marrow mononuclear cells (MNCs) were fractionated into four populations according to NS-GFP intensity. **(b)** MNC GFP mRNA expression level. **(c)** MNC NS mRNA expression level. **(d)** Bone marrow LSK cells were fractionated into four populations according to NS-GFP intensity. **(e)** LSK GFP mRNA expression level. **(f)** LSK NS mRNA expression level. Data are presented as the mean ± SD of the ratios of GFP or NS to β-actin mRNA copy numbers in fractionated cells (n = 3); ND, not detected.

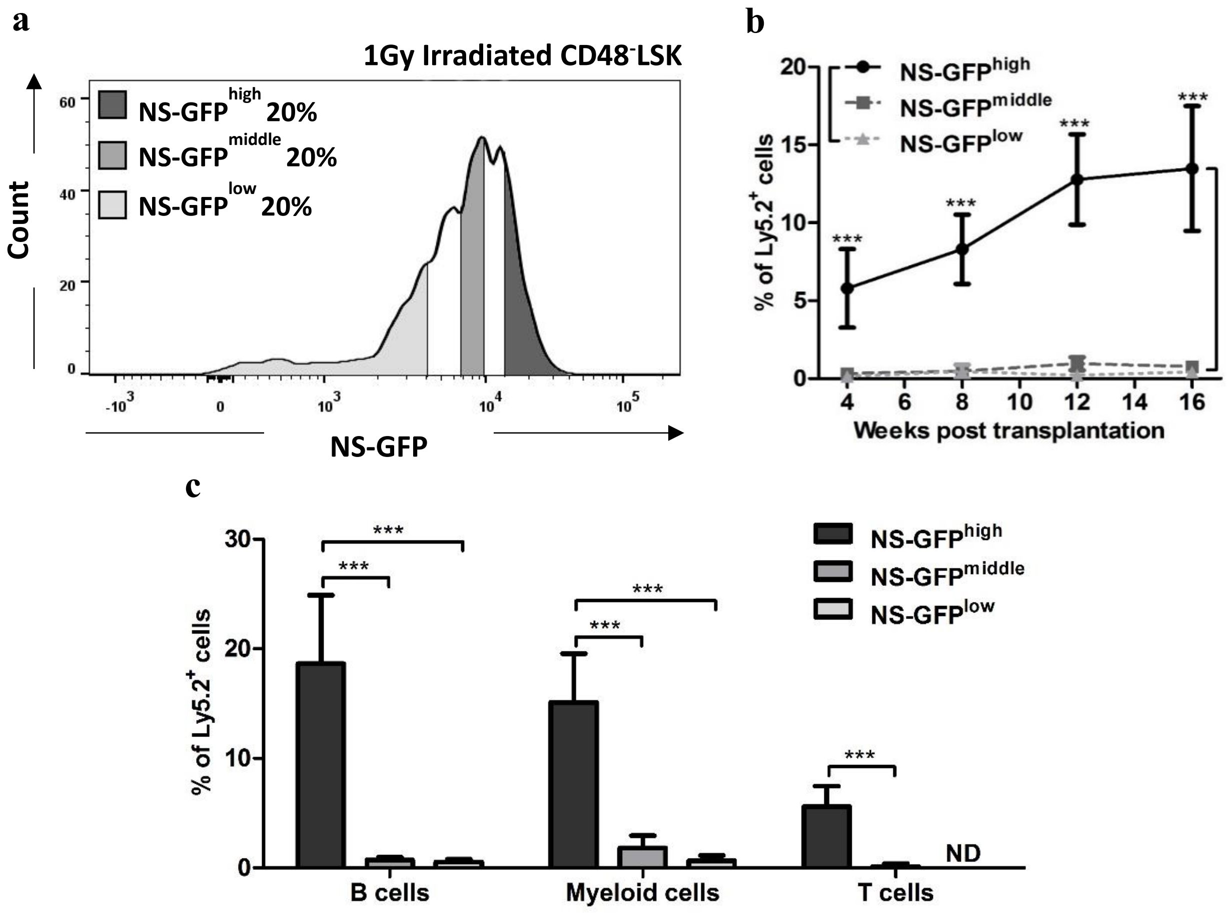

Supplementary Figure 2. Repopulating capacity of HSCs after radiation. (a) FACS was used to separate 1 Gy–irradiated bone marrow CD48^{-}LSK cells into five fractions according to NS-GFP intensity, of which three fractions—the top 20% (NS-GFP^{high}), middle 20% (NS-GFP^{middle}), and bottom 20% (NS-GFP^{low})—were used in subsequent experiments. 200 cells were transplanted per mouse. **(b)** Only the NS-GFP^{high} fraction of cells shows long-term reconstitution capacity. Data shown are the mean frequencies of Ly5.2^{+} cells in the peripheral blood ± SD (n = 5). **(c)** Lineage analysis of transplanted fractions showing that only NS-GFP^{high} has multilineage chimerism 16 weeks after transplantation. Data shown are the mean frequencies of Ly5.2^{+} cells among B cells, T cells or myeloid cells ± SD (n = 5). ***$p < 0.001$; ND, not detected.

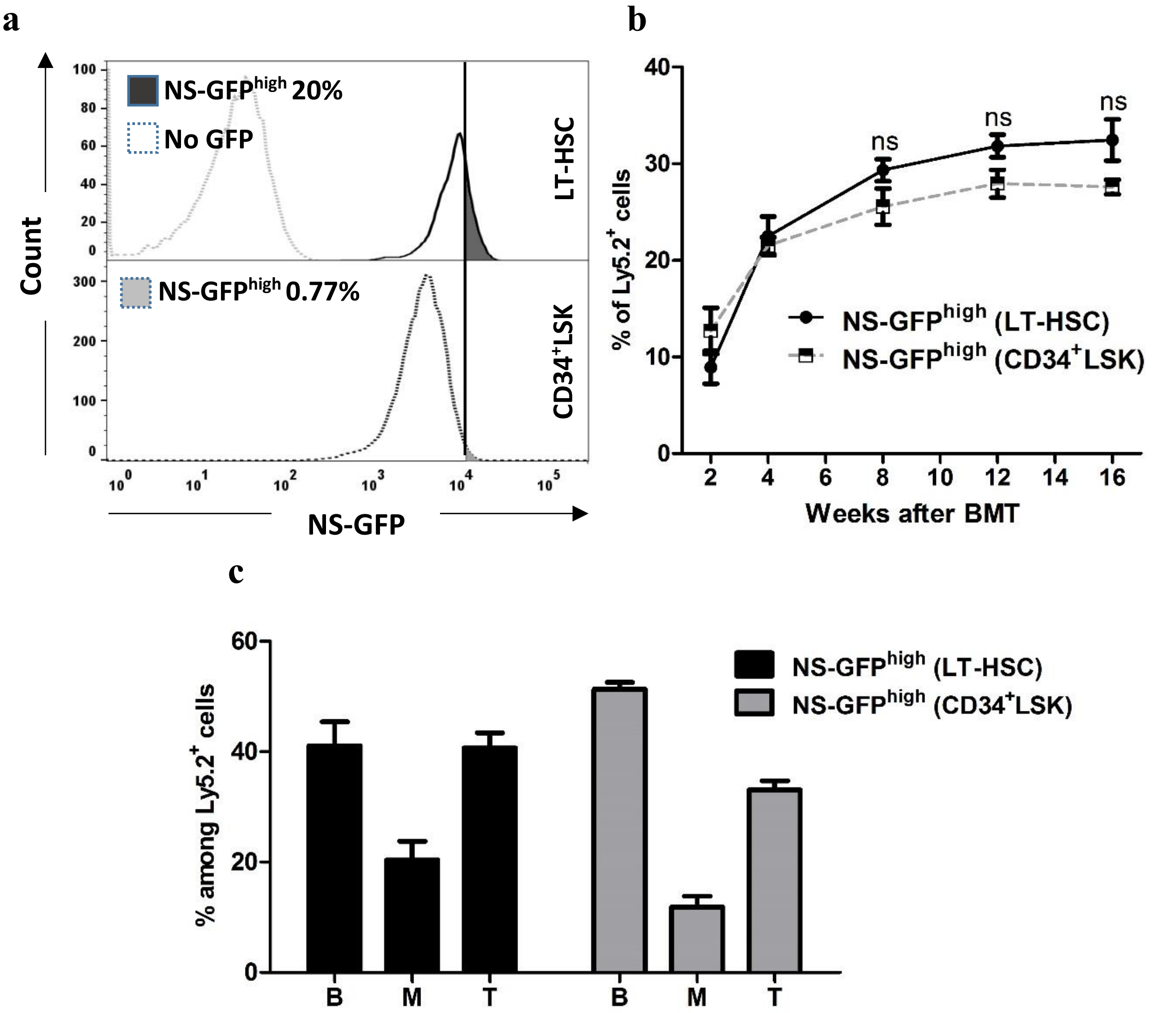

Supplementary Figure 3. Repopulating capacity of cells in the LT-HSC and CD34$^+$LSK compartments highly expressing NS-GFP. (a) FACS pattern of sorting LT-HSCs and CD34$^+$ LSK cells with high levels of NS-GFP intensity. **(b)** The transplanted fractions show comparable total repopulation capacity in peripheral blood. Ten cells were transplanted per mouse. Data shown are the mean frequencies of Ly5.2$^+$ cells in the peripheral blood ± SD (n = 5). **(c)** Lineage analysis of transplanted cells 16 weeks after transplantation showing comparable multilineage chimerism. Data shown are the mean frequencies of B cells, T cells or myeloid cells among Ly5.2$^+$ cells ± SD (n = 5). Note: The data of "NS-GFP^{high} (LT-HSC)" are identical to the "NS-GFP^{high} of LT-HSCs" data presented in Figure 6. ns; not significant. B; B cells. M; myeloid cells. T; T cells.
